# Predictors of antiretroviral therapy initiation: a cross-sectional study among Chinese HIV-infected men who have sex with men

**DOI:** 10.1186/s12879-015-1309-x

**Published:** 2015-12-15

**Authors:** Yu Liu, Yuhua Ruan, Sten H. Vermund, Chandra Y. Osborn, Pingsheng Wu, Yujiang Jia, Yiming Shao, Han-Zhu Qian

**Affiliations:** Vanderbilt Institute for Global Health, Vanderbilt University School of Medicine, Nashville, Tennessee USA; State Key Laboratory of Infectious Disease Prevention and Control (SKLID), Collaborative Innovation Center for Diagnosis and Treatment of Infectious Diseases, Chinese Center for Disease Control and Prevention (China CDC), Beijing, China; Department of Pediatrics, Vanderbilt University School of Medicine, Nashville, Tennessee USA; Department of Medicine, Vanderbilt University School of Medicine, Nashville, Tennessee USA; Department of Biomedical Informatics, Vanderbilt University School of Medicine, Nashville, Tennessee USA; Department of Biostatistics, Vanderbilt University School of Medicine, Nashville, Tennessee USA

**Keywords:** HIV, antiretroviral therapy, Continuum of care, Men who have sex with men, China

## Abstract

**Background:**

Early antiretroviral therapy (ART) initiation is crucial to achieve HIV viral suppression and reduce transmission. HIV-infected Chinese men who have sex with men (MSM) were less likely to initiate ART than other HIV-infected individuals. We assessed predictors of ART initiation among Chinese MSM.

**Methods:**

In 2010–2011, a cross-sectional study was conducted among MSM in Beijing, China. We examined ART initiation within the subgroup who were diagnosed with HIV infection prior to participation in the survey. Logistic regression models were fitted to evaluate socio-demographic and behavioral factors associated with ART initiation. The eligibility criterion in the 2010/2011 national HIV treatment guidelines was CD4 cell count <350 cells/μL or World Health Organization (WHO) clinical stage III/IV.

**Results:**

Of 238 eligible HIV-infected participants, the median duration of HIV infection was 15 months (range: 31 days-12 years); 62 (26.1 %) had initiated ART. Among 103 men with CD4 counts <350 cells/μL, 38 (36.9 %) initiated ART. Being married to a woman (adjusted odd ratios [aOR]: 2.50; 95 % confidence interval [CI]: 1.07-5.87), longer duration of HIV infection (aOR: 10.71; 95 % CI: 3.66-31.32), and syphilis co-infection (aOR: 2.58; 95 % CI: 1.04-6.37) were associated with a higher likelihood of ART initiation. Of 135 men with CD4 count ≥350 cells/μL, 24 (18 %) initiated ART. Being married to a woman (aOR: 4.21; 95 % CI: 1.60-11.06), longer duration of HIV infection (aOR: 22.4; 95 % CI: 2.79-180), older age (aOR: 1.26; 95 % CI: 1.1-1.44), Beijing *Hukou* (aOR: 4.93; 95 % CI: 1.25-19.33), presence of AIDS-like clinical symptoms (aOR: 3.97; 95 % CI: 1.32-14.0), and history of sexually transmitted infections (aOR: 4.93; 95 % CI: 1.25-19.43) were associated with ART initiation. Compared with men who did not initiated ART, those with ART were more likely to receive counseling on benefits of ART (96.8 % vs. 66.4 %, *P* = 0 < 0.01), HIV stigma coping strategy (75.8 % vs. 65.9 %, *P* = 0.04), mental health (66.1 % vs. 52.9 %, *P* = 0.02), and substance use (46.7 % vs. 36.6 %, *P* = 0.04).

**Conclusions:**

We documented low rates of ART initiation among Chinese MSM. Policy changes for expanding ART eligibility and interventions to improve the continuum of HIV care are in progress in China. Impact evaluations can help assess continuing barriers to ART initiation among MSM.

## Background

Antiretroviral therapy (ART) use among HIV-infected individuals is crucial for long-term success in reducing HIV transmission and disease progression [1, 2] Early initiation of ART can suppress viral replication and reduce harmful immune activation, slowing pathogenesis, and stem the spread of the virus (‘treatment-as-prevention’) [3, 4]. Mathematical modeling and observational studies suggested that the regions with higher ART coverage had reduced HIV transmission between regular and casual sexual partners, lower community viral load, and decreased HIV incidence [5–8]. The randomized clinical trial HPTN052 confirmed that early initiation of ART could significantly reduce sexual transmission of HIV among serodiscordant couples [9].

Despite these benefits of modern ART [2, 8, 10], many HIV-infected individuals in China are still not on ART [11]. A 2011 study showed that only 41 % (88,185/214,714) HIV-infected individuals had initiated ART [12], though the rate has been increasing steadily in the recent years. Chinese men who have sex with men (MSM) are less likely to start ART compared with other risk groups [13].

HIV epidemic among Chinese MSM is the most robust in all high risk groups [14–16]. According to the latest national estimates, HIV prevalence among Chinese MSM uniquely represented a marked uptrend (7.3 % in 2013 vs. 0.9 % in 2003), while in other risk groups it either dropped or maintained at a low level [17]. Male-to-male transmission accounted for 21.4 % of all new infections in 2013, with the fastest increasing rate among all risk groups [17]. As Chinese MSM may marry a women due to social and family pressures [18–20], HIV-infected MSM are also likely transmit the virus to their female sexual partner. Therefore, it is a crucial strategy for HIV prevention to expand ART coverage among Chinese MSM. However, there is little knowledge about ART initiation among Chinese MSM. A qualitative study suggested that Chinese MSM may not seek HIV care or initiate ART due to psychological, social, cultural and institutional barriers [21]. We conducted the first quantitative assessment of ART initiation and its predictors among Chinese HIV-infected MSM.

## Methods

### Study design and population

In 2010–2011, we conducted a cross-sectional study among Chinese MSM with a primary objective of assessing the association between male circumcision and HIV risk among MSM in Beijing, China. This study was described elsewhere [22–24]. In brief, participants were recruited from HIV/sexually transmitted disease (STD) clinics or the community, and through peer-referral; participants who were tested HIV positive from other studies in Beijing were also recruited. Eligibility criteria included: being a male, living in Beijing, self-reporting ever having sex with men, willingness to provide blood samples for HIV and syphilis serological tests and anal swab specimens for HPV testing, and willingness to provide written informed consent were eligible to participate in the study. All eligible participants were asked to complete a questionnaire interview, undertake genital examinations, and provide blood samples [24]. The study protocol was approved by the Institutional Review Boards of the National Center for AIDS/STD Control and Prevention of the Chinese Center for Disease Control and Prevention, and the Vanderbilt University School of Medicine.

### Data collection

Each participant completed a questionnaire on socio-demographic characteristics: age, ethnicity, marital status, education, occupation, registered household in Beijing (or Beijing *Hukou*), duration of living in Beijing, and sexual orientation. The questionnaire also assessed behavioral risk factors: unprotected insertive or receptive anal sex, most recent CD4+ T-lymphocyte cell count (CD4+ count), and history of sexually transmitted diseases (STD, including chlamydia, gonorrhea, syphilis, herpes simplex type 2, etc.). Those who had been diagnosed with HIV infection prior to participation in this study were also asked about AIDS-like clinical symptoms, experiences of using ART and HIV care and counseling, such as substance use, mental health, stigma coping strategies, benefits of ART, and prevention of HIV transmission.

### Laboratory testing

Enzyme-linked immunosorbent assay (ELISA, Wantai Biological Medicine Company, Beijing, China) was used for HIV-1 screening. Positive samples were confirmed by HIV-1/2 Western blot (HIV Blot 2.2 WB™; Genelabs Diagnostics, Singapore). Syphilis serology was determined through rapid plasma reagin (RPR, Shanghai Kehua Biotechnology Ltd, China) and confirmed by *Treponema pallidum* Enzyme-linked immunosorbent assay (TP-ELISA, Beijing Wantai Biological Pharmacy Enterprise Co., Ltd, China)[24].

### Statistical analysis

A significant portion of participants had been previously diagnosed with HIV infection, and were asked for ART use. Therefore, we performed a subgroup analysis among these participants to assess their ART initiation and its predictor variables.

We expressed descriptive statistics as proportions for categorical variables and as means and standard deviations, or medians and interquartile ranges for continuous variables, depending on the distribution of the variables. The socio-demographic, clinical, and behavioral characteristics were compared between those who had initiated ART and who had not in the whole study sample and in subgroups by CD4 count of 350 cells/uL, which was the cut point of ART eligibility in Chinese national guidelines during 2010 and 2011.We also examined WHO HIV clinical staging (III or IV) for dividing the subgroups. Continuous variables were compared with Wilcoxon rank-sum tests, and categorical variables were compared with the Chi-square or Fisher’s exact tests. Proportions of MSM receiving HIV care counseling services between men who did and did not initiate ART were compared using Chi-square tests.

To further assess the strength and direction of the associations between selected predictors and ART initiation stratified by CD4+ count, variables moderately associated with ART initiation (*P* < 0.2) in bivariable analyses were selected for multivariable logistic regression analyses. In the multivariable logistic regression models, variables with a *P* < 0.2 in the bivariable logistic regression analyses were preliminarily fitted into each model, respectively, then a manual backward selection procedure was used to conclude the final multivariable models that only retained covariates significantly associated with the outcome(*P* < 0.05). However, factors considered as *a priori* predictors or confounders based on prior evidence were forced to retain in the final models regardless of *P*-value [25]. All statistical analyses were performed using Stata 12.0™ (StataCorp LP, College Station, Texas, USA).

## Results

### Characteristics of Study Population

Among 1155 participants in the study, 290 were confirmed to be HIV positive (25.1 %), including 48 men newly diagnosed upon enrolled in the current study and 242 previously diagnosed. The 48 newly diagnosed HIV cases were excluded in the analysis since they had had no opportunity to initiate ART upon the survey. Of the 242 previously diagnosed HIV-infected MSM, 4 had missing data on ART initiation, and therefore, 238 (98.3 %) were included in this analysis.

The median age of these 238 men was 31 years (interquartile range [IQR], 26–37) and median duration from HIV diagnosis to participation in this survey was 15 months (IQR, 6–31).A majority of participants were Han ethnics (94.1 %), were currently unmarried (71.4 %) and employed (74.8 %), had college education (53.8 %), and had Beijing *Hukou* (61.8 %).

Over a quarter (26.1 %) men had started ART. Compared with those who had not initiated ART, men who had initiated ART were more likely (*P* < 0.05) to be older, be married to a woman, have Beijing *Hukou*, live longer in Beijing, have a longer duration of HIV-infection, experience adverse clinical symptoms, have a history of STD, and be co-infected with syphilis. However, those who had initiated ART were less likely (*P* < 0.05) to be engaged in unprotected insertive or receptive anal sex (Table 1).

**Table 1:** Sociodemographic and behavioral characteristics of HIV-infected men who have sex with men in Beijing, China

		Having initiated ART		
Characteristic	Total sample (N=238), n (%)	Yes (N=62), n (%)	No (N=176), n (%)	*P*-value
Age, median (IQR) (year)	31 (26–37)	36 (31–42)	30 (26–35)	<0.001
Ethnicity				0.69
Han	224 (94.1)	59 (95.2)	165 (93.7)	
Others	13 (5.9)	3 (4.8)	6.3 (6.3)	
Marital status				<0.001
Currently unmarried	170 (71.4)	31 (50.0)	139 (79.0)	
Currently married	68 (28.6)	31 (50.0)	37 (21.0)	
Education (year of schooling)				0.78
College or above (>12)	128 (53.8)	31 (50.0)	97 (55.1)	
Senior high school (10–12)	69 (29.0)	18 (29.1)	51 (29.0)	
Junior high school (7–9)	34 (14.3)	11 (17.7)	23 (13.1)	
Primary school (<=6)	7 (2.9)	2 (3.2)	5 (2.8)	
Occupational status				0.27
Employed	178 (74.8)	47 (75.8)	131 (74.4)	
Non-employed/retired	34 (14.3)	12 (19.4)	22 (12.5)	
Student	10 (4.2)	1 (1.6)	9 (5.1)	
Other	16 (6.7)	2 (3.2)	14 (8.0)	
Beijing *Hukou*				0.003
No	91 (38.2)	14 (22.6)	77 (43.7)	
Yes	147 (61.8)	48 (77.4)	99 (56.3)	
Duration of living in Beijing (year)				0.008
<5	99 (41.6)	17 (27.4)	82 (46.6)	
≥5	139 (58.4)	45 (72.6)	94 (53.4)	
Time from HIV infection to survey (month)				0.001
<15	115 (48.3)	6 (9.7)	109 (61.9)	
≥15	123 (51.7)	56 (90.3)	67 (38.1)	
Self-report health condition				0.91
Very good/good	193 (81.1)	50 (80.6)	143 (81.3)	
Somewhat poor/very poor	45 (18.9)	12 (19.4)	33 (18.7)	
Ever had AIDS-like clinical symptoms^a^				0.02
No	177 (74.4)	39 (62.9)	138 (78.4)	
Yes	61 (25.6)	23 (37.1)	38 (21.6)	
Had unprotected insertive anal sex with men in past 6 months				0.004
No	77 (71.3)	21 (95.5)	56 (65.1)	
Yes	31 (28.7)	1 (4.5)	30 (34.9)	
Had unprotected receptive anal sex with men in past 6 months				<0.001
No	86 (64.7)	27 (93.1)	59 (56.7)	
Yes	47 (35.3)	2 (6.9)	45 (43.6)	
Had multiple concurrent male partners in past 12 months				0.60
No	211 (90.9)	57 (93.4)	154 (90.1)	
Yes	21 (9.1)	4 (6.6)	17 (9.9)	
Had commercial sex in past 12 months				0.52
No	220 (94.4)	60 (96.8)	160 (93.6)	
Yes	13 (5.6)	2 (3.2)	11 (6.4)	
History of sexually transmitted diseases				0.003
No	100 (44.4)	17 (28.3)	83 (50.3)	
Yes	125 (55.6)	43 (71.7)	82 (49.7)	
Syphilis co-infection				0.003
No	93 (39.1)	15 (24.2)	78 (44.3)	
Yes	145 (60.9)	47 (75.8)	98 (55.7)	

Note: Sample size for each variable may vary due to missing data; *IQR* interquartile range, *ART* antiretroviral therapy

^a^Ever had at least one of these adverse clinical symptoms in the past 6 months: severe weight loss (>10 %), fever (>1 month, continuing or intermittent), chronic diarrhea (>1 month), severe bacterial infection (e.g. pneumonia), oral candidiasis, oral leukoplakia, tuberculosis other stage III or stage IV symptoms

Among 103 MSM with CD4 + cell count (CD4 count) <350 cells/μL who met the eligibility criteria for Chinese free ART program during this survey, 36.7 % initiated ART. Those who had initiated ART were more likely (*P* < 0.05) to be married to a woman, have longer duration of HIV-infection, and have syphilis co-infection, but were less likely (*P* < 0.05) to have unprotected insertive or receptive anal sex (Table 2).

**Table 2:** Sociodemographic and behavioral characteristics of HIV-infected men who have sex with men by CD4 count in Beijing, China

	CD4 count <350 cells/μL^a^				CD4 count ≥350 cells/μL			
Characteristics	Total (N=103), n (%)	Initiated ART (N=38), n (%)	Not initiated ART (N=65), n (%)	*P*-value	Total (N=135), n (%)	Initiated ART (N=24), n (%)	Not initiated ART (N=111), n (%)	*P*-value
Age (year)				0.07				<0.001
Median, IQR	32 (27–38)	34 (29–39)	30 (26–36)		31 (26–37)	41 (35–45)	30 (25–34)	
Ethnicity				1.00				0.59
Han	95 (92.2)	35 (92.1)	60 (92.3)		129 (95.6)	24 (100.0)	105 (94.6)	
Non-Han	8 (7.8)	3 (7.9)	5 (7.7)		6 (4.4)	0 (0)	6 (5.4)	
Marital status				0.03				<0.001
Currently unmarried	68 (66.0)	20 (52.6)	48 (73.8)		102 (75.6)	11 (45.8)	91 (82.0)	
Currently married	35 (34.0)	18 (47.4)	17 (26.2)		33 (24.4)	13 (54.2)	20 (18.0)	
Education (year of schooling)				0.92				0.52
College or above (>12)	57 (55.3)	21 (55.2)	36 (55.4)		71 (52.6)	10 (41.7)	61 (55.0)	
Senior high school (10–12)	24 (23.3)	8 (21.1)	16 (24.6)		45 (33.3)	10 (41.7)	35 (31.5)	
Junior high school (7–9)	19 (18.5)	8 (21.1)	11 (16.9)		15 (11.1)	3 (12.5)	12 (10.8)	
Primary school (≤6)	3 (2.9)	1 (2.6)	2 (3.1)		4 (3.0)	1 (4.1)	3 (2.7)	
Occupational status				0.87				0.39
Employed	81 (78.6)	31 (81.6)	50 (76.9)		97 (71.8)	16 (66.6)	81 (73.0)	
Non-employed/retired	15 (14.6)	5 (13.2)	10 (15.4)		19 (14.1)	7 (29.2)	12 (10.8)	
Student	2 (1.9)	1 (2.6)	1 (1.5)		8 (5.9)	0 (0)	8 (7.2)	
Other	5 (4.9)	1 (2.6)	4 (6.2)		11 (8.2)	1 (4.2)	10 (9.0)	
Beijing *Hukou*				0.19				<0.001
No	39 (37.9)	11 (28.9)	28 (43.1)		52 (38.5)	3 (12.5)	49 (44.1)	
Yes	64 (62.1)	27 (71.1)	37 (56.9)		83 (61.5)	21 (87.5)	62 (55.9)	
Duration of living in Beijing (year)				0.06				0.04
<5	45 (43.7)	12 (31.6)	33 (50.8)		54 (40.0)	5 (20.8)	49 (44.1)	
≥5	58 (56.3)	26 (68.4)	32 (49.2)		81 (60.0)	19 (79.2)	62 (55.9)	
Time from HIV infection to survey (month)				<0.001				<0.001
<15	46 (44.7)	5 (13.2)	41 (63.1)		69 (51.1)	1 (4.2)	68 (61.3)	
≥15	57 (55.3)	33 (86.8)	24 (36.9)		66 (48.9)	23 (95.8)	43 (38.7)	
Self-report health condition				1.00				0.53
Very good/good	77 (74.8)	28 (73.7)	49 (75.4)		116 (85.9)	22 (91.7)	94 (84.7)	
Somewhat poor/very poor	26 (25.4)	10 (26.3)	16 (24.6)		19 (14.1)	2 (8.3)	17 (15.3)	
Ever had AIDS-like clinical symptoms^b^				0.35				0.02
No	77 (74.8)	26 (68.4)	51 (78.5)		100 (74.1)	13 (54.2)	87 (78.4)	
Yes	26 (25.2)	12 (31.6)	14 (21.5)		35 (25.9)	11 (45.8)	24 (21.6)	
Had unprotected insertive anal sex with men in past 6 months				0.02				0.25
No	34 (79.1)	14 (100)	20 (69.0)		43 (66.2)	7 (87.5)	36 (63.2)	
Yes	9 (20.9)	0 (0)	9 (31.0)		22 (33.8)	1 (12.5)	21 (36.8)	
Had unprotected receptive anal sex with men in past 6 months				0.04				0.01
No	43 (75.4)	16 (94.1)	27 (67.5)		43 (56.6)	11 (91.7)	32 (50.0)	
Yes	14 (24.6)	1 (5.9)	13 (32.5)		33 (43.4)	1 (8.3)	32 (50.0)	
Had multiple concurrent male partners in past 12 months				0.71				1.00
No	93 (93.0)	36 (94.7)	57 (91.9)		118 (89.4)	21 (91.3)	97 (89.0)	
Yes	7 (7.0)	2 (5.3)	5 (8.1)		14 (10.6)	2 (8.7)	12 (11.0)	
Had commercial sex in past 12 months				1.00				0.35
No	94 (94.0)	36 (94.7)	58 (93.6)		126 (94.7)	24 (100.0)	102 (93.6)	
Yes	6 (6.0)	2 (5.3)	4 (6.4)		7 (5.3)	0 (0)	7 (6.4)	
History of sexually transmitted diseases				0.30				0.003
No	43 (43.0)	13 (35.1)	30 (47.6)		57 (45.6)	4 (17.4)	53 (52.0)	
Yes	57 (57.0)	24 (64.9)	33 (52.4)		68 (54.4)	19 (82.6)	49 (48.0)	
Syphilis co-infection				0.03				0.11
No	39 (37.9)	9 (23.7)	30 (46.2)		54 (40.0)	6 (25.0)	48 (43.2)	
Yes	64 (62.1)	29 (76.3)	35 (53.8)		81 (60.0)	18 (75.0)	63 (56.8)	

Note: Sample size for each variable may reduce due to missing data; *IQR* inter quartile range

^a^CD4 count <350 cells/μL was eligible for free antiretroviral therapy at the time of the study

^b^Ever had at least one of these adverse clinical symptoms in the past 6 months: severe weight loss (>10 %), fever (>1 month, continuing or intermittent), chronic diarrhea (>1 month), severe bacterial infection (e.g. pneumonia), oral candidiasis, oral leukoplakia, tuberculosis other stage III or stage IV symptoms

Of 135 MSM with CD4 count ≥350 cells/μL who did not meet the criteria, 17.8 % initiated ART. Those who had initiated ART were more likely (*P* < 0.05) to be older, married to a woman, have Beijing *Hukou*, live longer in Beijing, have longer duration of HIV-infection, have ever had clinical symptoms, have a history of STD, and have syphilis co-infection, but were less likely (*P* < 0.05) to have unprotected insertive or receptive anal sex (Table 2).

### Factors associated with ART initiation by CD4 count level

Table 3 shows the variables associated with ART initiation in both bivariable and multivariable logistic regression analyses. In the final multivariable logistic regression models, being married (adjusted odd ratio [aOR]: 2.50; 95 % confidence interval [CI]: 1.07-5.87), longer duration of HIV infection (aOR: 10.7; 95 % CI: 3.66-31.3), and syphilis co-infection (aOR: 2.58; 95 % CI: 1.04-6.37) were associated with a higher likelihood of ART initiation among subgroup of CD4 count <350 cells/uL. Being married (aOR: 4.21; 95 % CI: 1.60-11.06), longer duration of HIV infection (aOR: 22.42; 95 % CI: 2.79-180.01), older age (aOR: 1.26; 95 % CI: 1.1-1.44), Beijing *Hukou* (aOR: 4.93; 95 % CI: 1.25-19.33), presence of AIDS-like clinical symptoms (aOR: 3.97; 95 % CI: 1.32-14.0), and prior STD (aOR: 4.93; 95 % CI: 1.25-19.43) were associated with a higher likelihood of ART initiation among subgroup of CD4 count ≥350 cells/uL.

**Table 3:** Bivariable and multivariable logistic regression analyses of factors associated with initiation of antiretroviral therapy among HIV-infected men who have sex with men by CD4 count in Beijing, China

	CD4 count <350 cells/μL (n=103)^a^			CD4 count ≥350 cells/μL (n=135)		
Factors	Initiated ART, % (n=38)	Crude OR (95 % CI)	Adjusted OR (95 % CI)	Initiated ART, % (n=24)	Crude OR (95 % CI)	Adjusted OR (95 % CI)
Age in years; Median (IQR)	34 (29–39)	1.04 (0.99,1.09)*	1.02 (0.96,1.09)	41 (35–45)	1.18 (1.10,1.27)***	1.26 (1.11,1.44)***
Marital status						
Currently unmarried	29.4	1.00	1.00	10.8	1.00	1.00
Currently married	51.4	2.54 (1.09,5.91)**	2.50 (1.07, 5.87)**	39.4	5.38 (2.11,13.73)***	4.21 (1.60,11.06)***
Beijing *Hukou*						
No	28.2	1.00	1.00	5.8	1.00	1.00
Yes	42.2	1.86 (0.79,4.37)*	2.02 (0.74,5.50)	25.3	5.53 (1.56,19.63)***	4.93 (1.25,19.33)**
Duration of living in Beijing (year)						
<5	26.7	1.00	1.00	9.3	1.00	1.00
≥5	44.8	2.23 (0.96,5.17)*	1.91 (0.77, 4.75)	23.5	3.00 (1.05,8.61)**	1.98 (0.28,14.04)
Time from HIV infection to survey (month)						
<15	10.9	1.00	1.00	1.5	1.00	1.00
≥15	57.9	11.28 (3.88,32.78)***	10.71(3.66,31.32)***	34.9	36.37(4.74,279.22)***	22.4(2.79,180.01)***
Ever had AIDS-like clinical symptoms^b^						
No	33.8			13.0	1.00	1.00
Yes	46.2	NS	NS	31.4	3.07 (1.22,7.71)**	3.97 (1.32,13.96)**
Had unprotected insertive anal sex with men in past 6 months						
No	41.2	1.00	1.00	16.3		
Yes	0	-	-	4.6	NS	NS
Had unprotected receptive anal sex with men in past 6 months						
No	37.2	1.00	1.00	25.6	1.00	1.00
Yes	7.1	0.13 (0.02,1.09)*	0.22 (0.02,2.13)	3.0	0.09 (0.01,0.75)**	0.11 (0.01,1.40)
History of sexually transmitted diseases						
No	30.2			7.0	1.00	1.00
Yes	42.1	NS	NS	27.9	5.14 (1.63,16.16)***	4.93 (1.25,19.43)**
Syphilis co-infection						
No	23.1	1.00	1.00	11.1	1.00	1.00
Yes	45.3	2.76 (1.13,6.74)**	2.58 (1.04,6.37)**	22.2	2.29 (0.84,6.20)*	1.39 (0.41,4.72)

*IQR* inter quartile range, *OR* odds ratio, *CI* confidence interval, *NS* not selected for logistic regression analyses because *P*>0.2 in Table 2

^a^CD4 count <350 cells/μL was eligible for free antiretroviral therapy at the time of the study

^b^Ever had at least one of these clinical symptoms in the past 6 months: severe weight loss (>10 %), fever (>1 month, continuing or intermittent), chronic diarrhea (>1 month), severe bacterial infection (e.g. pneumonia), oral candidiasis, oral leukoplakia, tuberculosis other stage III or stage IV symptoms

*P<0.2, **P<0.05, ***P<0.01

### HIV care counseling services received

**Fig. 1:**
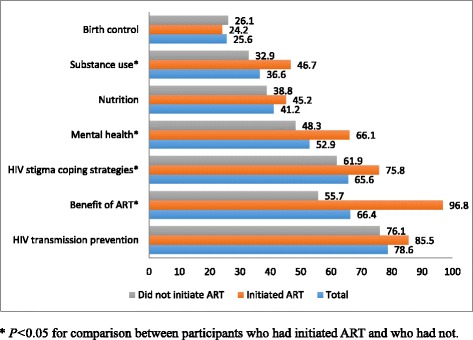
HIV care counseling services received among participants by ART initiation status

The top three HIV care counselling received by the participants were HIV transmission prevention (78.6 %), benefits of ART (66.4 %), and HIV stigma coping strategy (65.6 %). Other counseling topics included mental health (52.9 %), nutrition (41.2 %), substance abuse (36.6 %), and birth control (25.6 %) (Fig. 1). MSM who initiated ART were more likely to receive counseling than those without ART on benefits of ART (96.8 % vs. 66.4 %, *P* < 0.01), HIV stigma coping strategy (75.8 % vs. 65.9 %, *P* = 0.04), substance use (46.7 % vs. 36.6 %, *P* = 0.04), and mental health (66.1 % vs. 52.9 %, *P* = 0.02). There was no statistically significant difference in receiving counseling on HIV transmission prevention, birth control, and nutrition between those who had or had not initiated ART.

## Discussion

To our knowledge, this is the first study on predictors of ART initiation and receipt of HIV care among Chinese HIV-infected MSM. In the study, we found older age and ever having adverse clinical symptoms were positively associated with ART initiation, and this is consistent with findings from other regions [26]. Due to social and family pressure, some Chinese MSM are married with women or have female sexual partners [27]. Our study showed that married MSM were more likely to start ART than unmarried MSM. Married men might have more sense of responsibility for the health of themselves and their wives, and therefore have started ART; however, more research is needed to explore the reasons. Beijing *Hukou* and longer duration of living in Beijing were also positively associated with ART initiation. Migrant MSM, particularly recent migrants, might not know that free ART is for everyone, or where they could get it. It is suggested HIV intervention programs should give special emphasis among migrant MSM who account for the majority of MSM population in many large cities. In addition, Men who had initiated ART reported receiving more HIV care counseling services than those without ART, and therefore could get more health benefits from the counseling.

Our findings suggest considerations for the design of future intervention programs. The positive association between being married and ART initiation observed among both ART eligible and ineligible MSM (using criteria in place in 2010–2011) suggest that a built-in educational or psychological counseling session would be necessary for cultivating the sense of responsibility for spouses and family among married HIV-infected MSM, which might facilitate early ART initiation. Given that the longer time from HIV diagnosis to the survey and older age was positively associated with ART initiation, greater government and community efforts to target and engage men earlier and more intensively may be effective, alongside linking recently infected MSM to HIV care for ART initiation. The benefits of legal residence (Beijing *Hukou*) in accessing HIV care reflect both one’s ability to use local medical services and social benefits, and also one’s motivation and willingness in seeking these services. People without local residence might be more financially, physically, and/or emotionally burdened as compared to local residents. In our study, having Beijing *Hukou* played a crucial role in ART initiation among MSM who did not meet free ART criterion. In the meantime, those who had longer duration living in Beijing demonstrated a higher likelihood of proactive ART initiation. Thus, our findings suggest the need for researching the sexual network and cooperating with local gay organizations with outreach to the migrant MSM who recently move to Beijing and/or are transiently working in Beijing.

China started its national free ART program in 2003 [28]. This program has significantly reduced mortality among HIV-infected individuals [29, 30]. However, our study showed that only 26 % HIV-infected participants who had been infected with HIV for a median 15 months (IQR, 6–31) initiated ART, and the rate was lower than other risk groups in China [31–33] and MSM in other countries [34–38]. Men who met treatment criteria (36.7 %) were more likely to initiated ART than those who did not (17.8 %), but both were quite low. Our study examined ART initiation among Chinese for the first time. Previous surveys suggested considerable willingness to receive ART [13, 39], yet this willingness seems not translating into uptake of ART, where studies revealed low ART use [14, 40–42]. There are multiple reasons for low ART use. First, Chinese free ART program were originally developed to provide “Four Free and One Care” (free ARV drugs, free prevention of mother-to-child transmission, free voluntary counseling and testing, free schooling for children orphaned by AIDS, and care to people living with HIV/AIDS) to HIV-infected former plasma donors and persons who injected drugs and their family members. Chinese government has vigorously promoted ART use in these populations [43]. In contrast, the promotion activities of ART use among HIV-infected MSM were sporadic prior to 2011, mainly through programs funded by international agencies such as Global Fund for AIDS and Gates Foundation. HIV-infected MSM might not be aware that they can also use the Chinese free ART program. Second, the HIV epidemic among Chinese MSM is more recent compared with those in other risk groups. Although prevention-oriented intervention programs exist for Chinese MSM, ART is relatively new and therefore unfamiliar to many [44]. Third, the majority of HIV-infected MSM are generally young (<40 years), well educated, and living in large cities, so they are well accessible to HIV-related knowledge and information. They are concerned about side-effects of first-line generic ARVs in the Chinese free ART program and possible drug resistance due to failure in treatment adherence; they are concerned about no alternative drugs due to the limited number of available antiretroviral drugs (ARVs) in Chinese ART program [45–47]. Fourth, Chinese MSM face dual stigma of HIV and homosexuality, and they may encounter discrimination from the community and healthcare providers, which prevents them from seeking HIV care and treatment [48–50]. Last, the stricter eligibility criteria in earlier years might also exclude some MSM from treatment. China ART program relaxed its criteria to CD4 count <500 cells/μL in 2013. Actually in some cities, HIV-infected individuals can start ART immediately at diagnosis, like do in high income countries [51–53].

We recognized the limitations of our study: (1) With a relatively small sample of HIV-infected Chinese MSM living in Beijing, findings may not represent very precise estimates or be generalizable to other settings; (2) Data on socio-demographic characteristics, risky behaviors, and some clinical factors were based on self-reporting, subjecting these data to recall and social desirability biases; (3) In this cross-sectional study, we were not able to ascertain some key information, such as the exact time of ART initiation, and viral load or other markers for viral replication or immune activation at HIV diagnosis or ART initiation. These factors might also affect the physician’s decision on prescribing ART. However, the main criteria for ART initiation are based on CD4 count and WHO clinical staging, but not on HIV viral load according to Chinese ART protocol.

## Conclusions

In conclusion, the rates of ART initiation among Chinese MSM were low as of 2011. Compared to other subgroups, HIV-infected Chinese MSM are less likely to initiate ART. This is the first study in China to investigating factors associated with ART initiation in this marginalized population. Policy changes for expanding ART eligibility and interventions to improve the continuum of HIV care are in progress in China. Impact evaluations can help assess continuing barriers to ART initiation among MSM.

## Abbreviations

ART: Antiretroviral therapy
ARV: Antiretroviral
MSM: Men who have sex with men
WHO: World Health Organization
STD: Sexually transmitted diseases
ELISA: Enzyme-linked immunosorbent assay
WB: Western blot
RPR: Rapid plasma reagin
